# Prime Editing Permits the Introduction of Specific Mutations in the Gene Responsible for Duchenne Muscular Dystrophy

**DOI:** 10.3390/ijms23116160

**Published:** 2022-05-31

**Authors:** Cédric Happi Mbakam, Joël Rousseau, Guillaume Tremblay, Pouiré Yameogo, Jacques P. Tremblay

**Affiliations:** Centre de Recherche du CHUQ-Université Laval, Québec, QC G1V4G2, Canada; cedric.happi-mbakam.1@ulaval.ca (C.H.M.); joel.rousseau@crchudequebec.ulaval.ca (J.R.); guillaume.tremblay.22@ulaval.ca (G.T.); pouire.yameogo.1@ulaval.ca (P.Y.)

**Keywords:** prime editing, CRISPR/Cas9, Duchenne Muscular Dystrophy, *DMD* gene, mutations

## Abstract

The Prime editing technique derived from the CRISPR/Cas9 discovery permits the modification of selected nucleotides in a specific gene. We used it to insert specific point mutations in exons 9, 20, 35, 43, 55 and 61 of the Duchenne Muscular Dystrophy (*DMD*) gene coding for the dystrophin protein, which is absent in DMD patients. Up to 11% and 21% desired mutations of the *DMD* gene in HEK293T cells were obtained with the PRIME Editor 2 (PE2) and PE3, respectively. Three repeated treatments increased the percentage of specific mutations with PE2 to 16%. An additional mutation in the protospacer adjacent motif (PAM) sequence improved the PE3 result to 38% after a single treatment. We also carried out the correction of c.428 G>A point mutation in exon 6 of the *DMD* gene in a patient myoblast. Myoblast electroporation showed up to 8% and 28% modifications, respectively, for one and three repeated treatments using the PE3 system. The myoblast correction led to dystrophin expression in myotubes detected by Western blot. Thus, prime editing can be used for the correction of point mutations in the *DMD* gene.

## 1. Introduction

Duchenne muscular dystrophy is an X-linked genetic disease which results from mutations in the *DMD* gene leading to a lack of dystrophin expression in muscle fibres [1]. The disease is mainly characterized by progressive muscle wasting and patients may die before the age of 30 because of cardiorespiratory complications [2,3]. The *DMD* gene contains 79 exons and is considered one of the biggest human genes with approximately a 2200 kb length [4]. Single or multi-exon deletions account for about 60 to 70% of cases. Point mutations represent around 26% of DMD cases and there are 10 to 15% exonic duplications [5]. Most of the point mutations are single nucleotide modifications resulting in a non-sense codon, blocking the dystrophin protein expression in muscle fibres [4].The rapid development of gene editing techniques derived from the CRISPR/Cas9 discovery now permits the modification of the human genome and opens the possibility of developing therapies for hereditary diseases [6,7]. In particular, the base editing technique [8] and the prime editing technique [9] permit the modification of a single targeted nucleotide. The base editing technology uses a Cas9 nickase (D10A) fused either with cytidine deaminase or adenine deaminase [8,10]. This system permits nicking the complementary strand to the Protospacer Adjacent Motif (PAM) and to chemically modify either a cytidine (C) into thymine (T) [8] and an adenine (A) into guanine (G) in a window of 5 nucleotides included in the protospacer sequence [10]. However, a cytidine (C) may also be modified into a guanine (G) [11], or into an adenine (A) [12]. The prime editing technology uses a Cas9 nickase (H840A) and a prime editing guide (pegRNA). This Cas9 nickase permits nicking the strand containing the PAM and this is fused through a flexible linker with reverse transcriptase (RT), which permits the synthesis of the DNA fragment containing the desired edit. The pegRNA includes the single guide sequence (sgRNA, a variable sequence of 20 nucleotides targeting a specific DNA sequence and a constant scaffold sequence) and a 3′ extension coding for the Reverse Transcriptase Template (RTT) and a Primer Binding Site (PBS). The RTT contains nucleotides which are complementary to the strand containing the PAM except for the nucleotide(s) to be modified [9].

In this article, we show that the prime editing technology can produce specific point mutations in the *DMD* gene. Since at the beginning of the project we did not have access to DMD patient cells containing non-sense mutation, we have initially mutated amino acid to produce non-sense mutations observed in DMD Canadian patients. We further corrected the c.428 G>A point mutation in exon 6 of *DMD* gene in a patient myoblasts. This confirmed that prime editing can precisely modify the *DMD* gene and lead to dystrophin expression.

## 2. Results

### 2.1. Mutation of Several DMD Exons by Prime Editing

**Figure 1:**
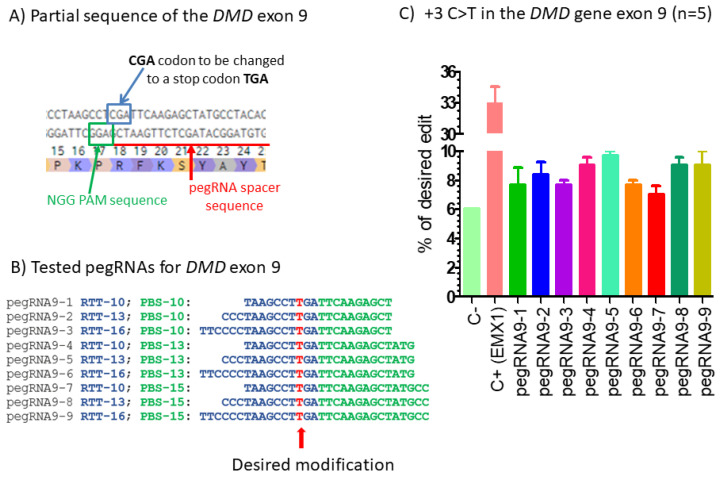
Prime editing of DMD exon 9. (**A**) Partial sequence of exon 9 of the *DMD* gene. The SpCas9n-RT PAM is in a green box and the pegRNA protospacer sequence is underlined in red. (**B**) The sequences 5′ to 3′ of the Primer Binding Site (PBS, in green) and of the Reverse Transcriptase Template (RTT, in blue) of 9 pegRNAs targeting DMD exon 9. The T in red in the RTT permits introducing an adenosine (**A**) in the strand containing the PAM (in the present case the antisense strand). This results in the presence of a thymine in the sense strand modifying the arginine codon (CGA) into a stop codon (TGA). (**C**) Results of Sanger sequencing and EditR analysis of prime editing of the DMD exon 9. There was a high background level (6%) of thymidine (T) in the position of the targeted nucleotide. However, pegRNA9-2 to pegRNA9-9 increased the percentage of T up to 10%. A control pegRNA-EMX1 targeting the EMX1 gene produced a 33% modification in that gene. The experiments were carried out in five independent replicates (*n* = 5).

**Figure 2:**
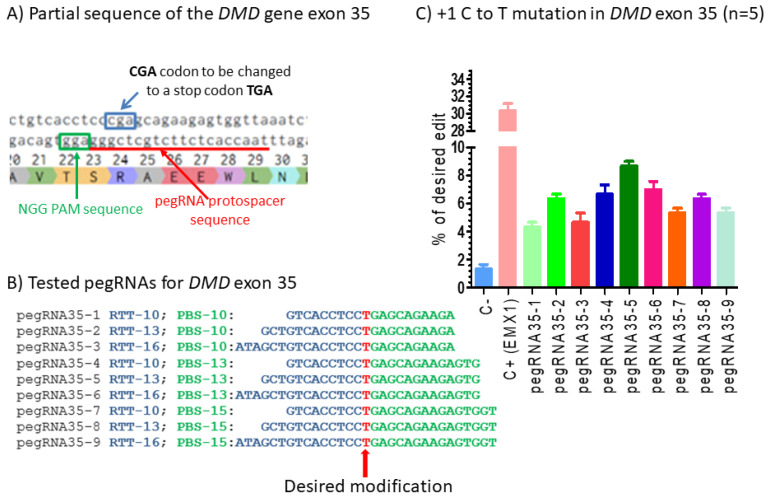
Prime editing of DMD exon 35. (**A**) Partial sequence of exon 35 of the *DMD* gene. The SpCas9n-RT PAM is in a green box and the pegRNA protospacer sequence is underlined in red. (**B**) The sequences (5′ to 3′) of the Reverse Transcriptase Template (RTT, in blue) and the Primer Binding Site (PBS, in green) of nine pegRNAs targeting DMD exon 35. The T in red in the RTT permits introducing an adenosine (**A**) in the strand containing the PAM (in the present case the antisense strand). This results in the presence of a thymine (T) in the sense strand, thus modifying the arginine codon (CGA) into a stop codon (TGA). (**C**) Results of Sanger sequencing and EditR analysis of prime editing of the DMD exon 35. There was a 2% background level of thymidine (T) in the position of the targeted nucleotide. However, several pegRNAs increased the percentage of T up to 8%. The control pegRNAs-EMX1 produced a 31% modification in that gene. These experiments were carried out in five independent replicates (*n* = 5).

To confirm that the prime editing technology can modify a specific nucleotide in the *DMD* gene, we first modified a single nucleotide in exon 9 of the *DMD* gene. We selected the SpCas9 H840A nickase because there was an NGG PAM (i.e., 5′AGG 3′) in the non-coding DNA strand close to the targeted cytidine nucleotide of an arginine codon (CGA) to be mutated into a thymine to create a TGA stop codon (Figure 1A). We tested nine different pegRNAs having different RTT and PBS sequences (Figure 1B). The pCMV-PE2 plasmid (Addgene #132775) coding for the SpCas9 H840A nickase fused with the M-MLV reverse transcriptase (M-MLV RT) and the pU6-pegRNA-GG-acceptor plasmid (Addgene #132777) coding for one of the pegRNAs under a U6 promoter were transfected in HEK293T cells. The RTT sequences of the different tested pegRNAs (sequence list in Figure 1B) produced the intended mutation. A pegRNA used in Anzalone et al. article [9] to mutate a nucleotide in the EMX1 gene was also used as a positive control. DNA was extracted from the cells 3 days later. The percentage of modifications of the targeted cytidine into thymine was analyzed by Sanger sequencing and EditR program (https://moriaritylab.shinyapps.io/editr_v10/ (accessed on 1 May 2022)) [13]. The pegRNAs-EMX1 targeting the EMX1 gene produced a 33% (Figure 1C) or 31% (Figure 2C) modification of the targeted nucleotide. Negative control cells were transfected only with a plasmid containing a reporter eGFP gene. The EditR analysis suggested that there was a 6% background presence of a T nucleotide at the position of the targeted C in exon 9 of the HEK293T cells (Figure 1C). This is probably due to the heterogeneity of the cell population for that gene sequence. However, when the cells were transfected with the pCMV-PE2 and each of the nine pegRNAs encoded in plasmid pU6-pegRNA-GG-acceptor (Addgene #132777), at the most only 10% of the targeted cytidine were thymidine in the post treatment sequences (Figure 1C). Given the background noise, this suggests that there were about only 4% real mutations of the targeted cytidine into thymidine with pegRNA9-5 and pegRNA9-9.

We also designed nine pegRNAs to induce a C to T mutation in an arginine codon CGA in DMD exon 35 to form a TGA stop codon (Figure 2A,B). Plasmids pU6-pegRNA-GG-acceptor coding for each of these nine pegRNAs under a U6 promoter were co-transfected in HEK293T cells with the pCMV-PE2 plasmid. Negative control cells were transfected with a plasmid containing a reporter eGFP gene. This permitted us to confirm a good level (at least 80%) of transfection. The DNA was extracted 3 days later, and the mutations were analyzed by Sanger sequencing and the EditR online program. There was only 2% of thymine in the cytidine targeted site in the negative control cells. The percentage of C to T mutations in the targeted arginine codon ranged from 4 to 8% for the different pegRNAs (Figure 2C). Given the presence of 2% background mutations in exon 35 in the HEK293T cells, this means up to 6% real targeted mutations.

**Figure 3:**
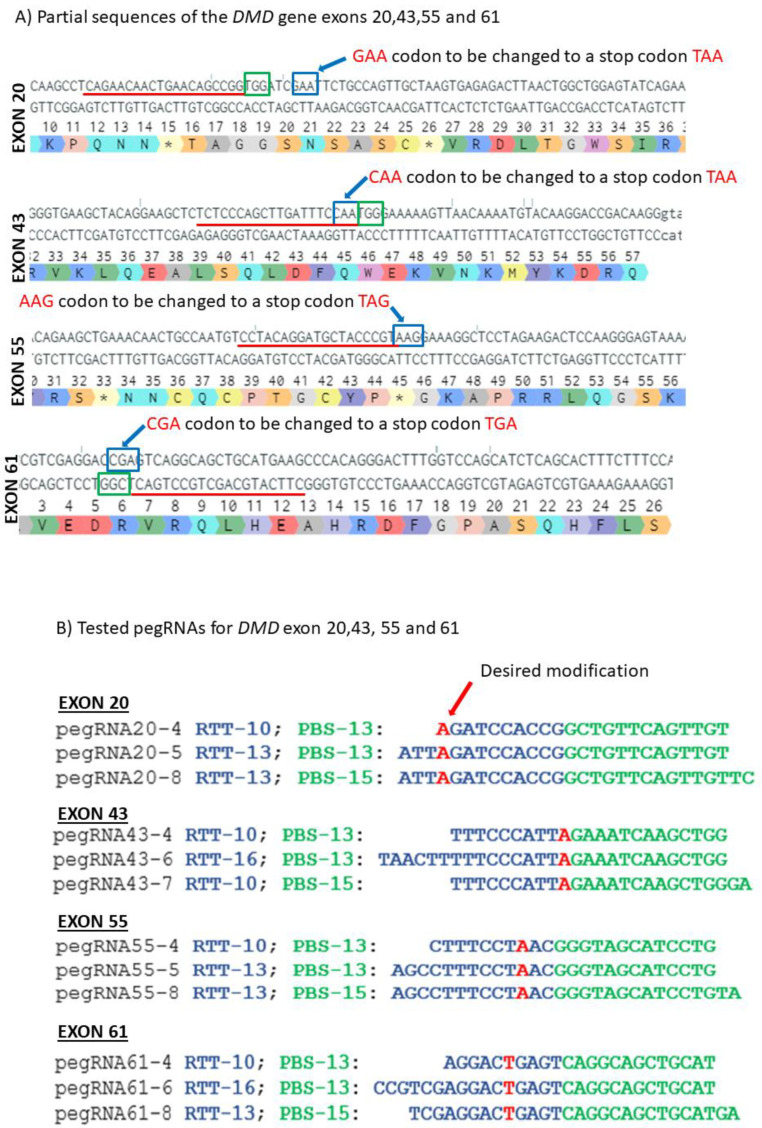

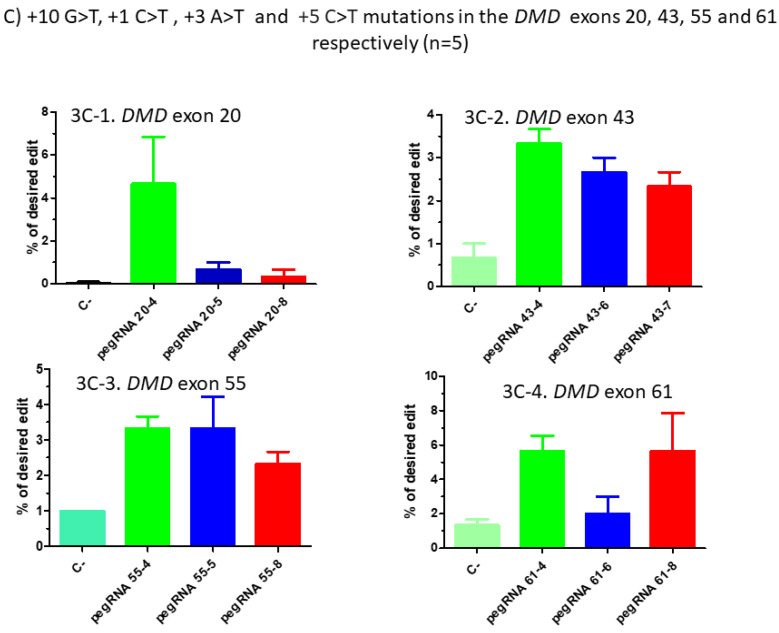
Prime editing of DMD exons 20, 43, 55 and 61. (**A**) Partial sequences of exons 20, 43, 55 and 61 of the *DMD* gene. The SpCas9n-RT PAMs are in a green box and the pegRNA protospacer sequences are underlined in red. The codons to be changed are in a blue box for each exon. (**B**) The sequences (5′ to 3′) of the Reverse Transcriptase Template (RTT, in blue) and of the Primer Binding Site (PBS, in green) of the three different pegRNAs targeting each of these exons of the *DMD* gene. The nucleotides in red colour in the RTT sequences represent the desired mutation to be introduced in the selected exons. (**C**) Results of Sanger sequencing and EditR analysis of prime editing of the DMD exons 20, 43, 55 and 61. For exon 20, there was 0% background level of thymidine (T) in the position of the targeted nucleotide. Results showed 4.5% of the desired edit with pegRNA20-4 and 1% with pegRNA20-5 and pegRNA20-8. For exon 43, there was a 1% background level of thymidine (T) in the position of the targeted nucleotide. The targeted modification showed 2.5% with pegRNA43-6 and pegRNA43-7 and 3.5% with pegRNA43-4. For exon 55, there was a 1% background level of thymidine (T) at the position of the desired edit. The targeted nucleotide showed 3.5% with pegRNA55-5 and pegRNA55-8 and 2.5% with pegRNA55-4. For exon 61, there was 1% background level of thymidine (T) in the targeted nucleotide position. The targeted modification showed 2% with pegRNA61-6 and 6% with pegRNA61-8 and pegRNA61-4. These experiments were carried out in five independent replicates (*n* = 5).

We also designed three different pegRNAs targeting each of the exons 20, 43, 55 and 61 of the *DMD* gene (Figure 3 A,B) to modify the glutamate codon GAA into the stop codon TAA, the glycine codon CAA into the stop codon TAA, the lysine codon AAG into the stop codon TAG and the arginine codon CGA into the stop codon TGA, respectively (Figure 3A). The pU6-pegRNA-GG-acceptor plasmid coding for each of the pegRNAs and the pCMV-PE2 plasmid coding for Cas9 nickase nuclease and the reverse transcriptase enzyme were co-transfected in HEK293T. DNA was extracted, amplified by PCR and sequenced by the Sanger method. The EditR analysis showed that the G to T mutation in exon 20 ranged from 1 to 9% with 0% background in HEK293T cells negative control. The negative control in exons 43 and 55 showed only 1% background mutations in HEK293T cells. There were only 2 to 4% additional mutations with different pegRNAs. The desired editing percentage for exon 61 ranged from 3 to 10% with 1% background T in the position of the edit.

### 2.2. Improving Prime Editing Percentages

#### 2.2.1. Repeated Treatments

**Figure 4:**
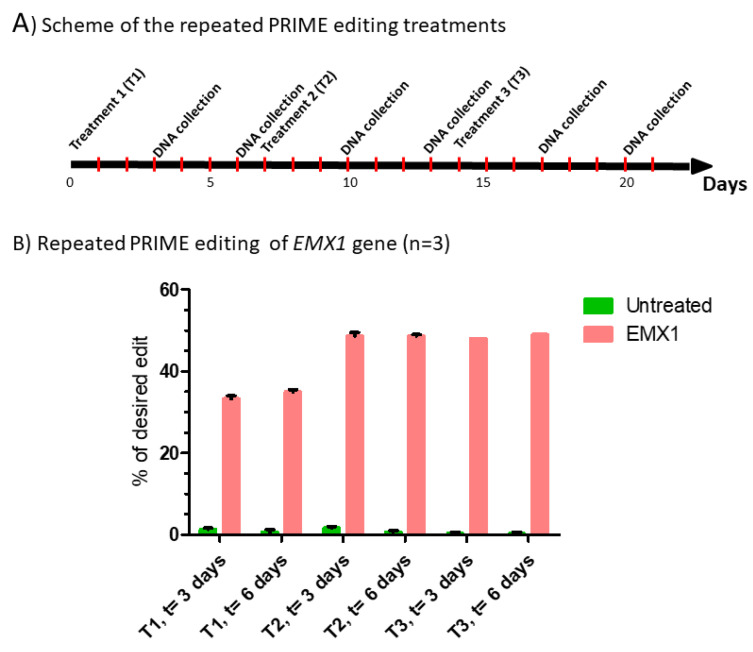

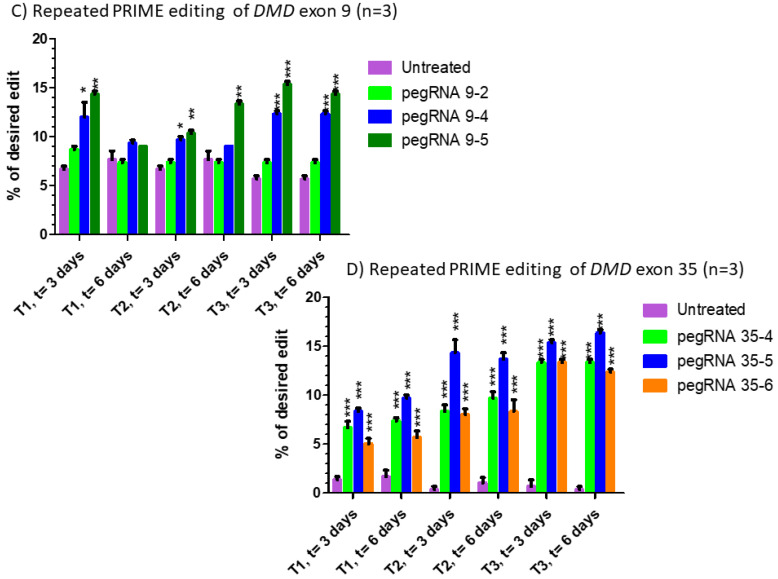
Repeated prime editing (**A**) Scheme summarizing the timing of the three successive prime editing treatments. The experiment was done in triplicate. HEK293T cells were transfected three times (at days 0, 7 and 14) with the plasmids pCMV-PE2 and pU6-pegRNA-GG-acceptor. The pegRNAs were targeting either the EMX1 gene (**B**), exon 9 of the *DMD* gene (**C**) or exon 35 of the *DMD* gene (**D**). DNA was extracted from cell samples at 3 and 6 days after each treatment (i.e., at days 3, 6, 10, 13, 17 and 20). The percentages of desired nucleotide mutations were determined by Sanger sequencing and analysis with EditR. The figures illustrate the average and standard deviations. These percentages increased with the second treatment for the EMX1 gene and DMD exon 35. The third treatment with pegRNA35-5 and pegRNA35-6 increased the mutation of DMD exon 35. The results were analyzed by an analysis of variance. The *, ** and *** indicate, respectively, a level of significance with a p value less than 0.001, 0.0001 and 0.00001 relative to the untreated cells (negative control). These experiments were carried out in independent triplicates (*n* = 3).

Since the percentage of targeted mutations was low, we tested different methods to increase the editing. We initially tested the hypothesis that the percentage of editing could be increased by multiple successive prime editing treatments (Figure 4A). We selected the three best pegRNAs identified in Figure 1C (pegRNA9-2, pegRNA9-4 and pegRNA9-5) and 2C (pegRNA35-4, pegRNA35-5 and pegRNA35-6). For the first treatment (T1), HEK293T cells were transfected with the pCMV-PE2 plasmid and a plasmid coding for a pegRNA targeting the EMX1 gene (positive control) or the *DMD* gene (exon 9 or 35). DNA was extracted from cell samples 3 and 6 days after the T1 treatment. Three days after this first treatment, the pegRNA targeting the EMX1 gene produced a 33% modification of the targeted nucleotide (Figure 4B). Considering the presence of 8% background mutation in exon 9 in the negative control, the pegRNAs targeting the DMD exon 9 produced only 1 to 7% additional mutations of the targeted cytidine into thymine after this first treatment (Figure 4C). For the pegRNAs targeting exon 35 of the *DMD* gene, the first prime editing treatment produced 5% to 8% modifications of the targeted C into a T (Figure 4D). However, the background noise was only 1 to 2% in this exon with negative controls. The percentages of mutations of the EMX1 and *DMD* genes remained relatively the same in samples obtained at 3 or 6 days after the first treatment (Figure 4B,C,D). For the second treatment (T2), some cells from which the DNA was not extracted were re-transfected the next day with the pCMV-PE2 plasmid and a plasmid coding for the same pegRNA. DNA was extracted 3 and 6 days after this second treatment (T2). The percentage of EMX1 mutations increased to 49% (Figure 4B). The percentage of mutation of the targeted cytidine also increased with some pegRNAs (especially for pegRNA35-5), which reached 14% 3 days after the second treatment (Figure 4D). However, the percentage of mutations induced by pegRNA9-2 targeting DMD exon 9 did not increase (Figure 4C). The mutagenic treatment was repeated a third time (T3) on the remaining cells and DNA was extracted again 3 and 6 days later. The percentage of mutations of the EMX1 gene did not increase further and remained at 49% (Figure 4B). However, the percentage of mutations in the DMD exon 35 increased again for pegRNA35-4 and pegRNA35-6, reaching 16% (Figure 4D). The percentage of mutations induced by pegRNA9-2 did not increase with repeated treatment.

#### 2.2.2. Inducing a Second Nick: The PE3 Method

**Figure 5:**
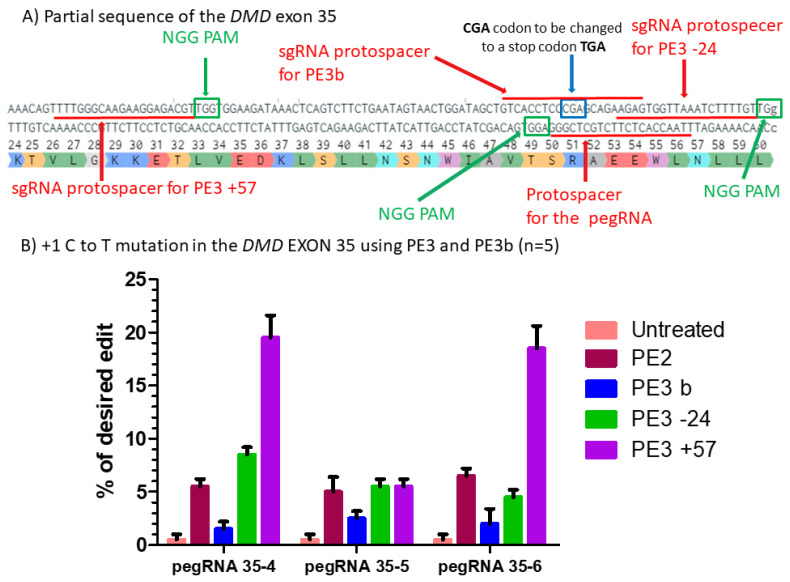
Prime editing of DMD exon 35 using the PE3 and PE3b strategies. (**A**) Partial sequence of exon 35 of the *DMD* gene including the sgRNA protospacer sequences for PE3. The SpCas9n PAM sequences are in green boxes and the pegRNA protospacer sequences are underlined in red. (**B**) Results of Sanger sequencing and EditR analysis of prime editing of the DMD exon 35 for PE3 and PE3b strategies. There was a 1% background level of thymidine (T) in the position of the targeted nucleotide. However, pegRNA35-4 and pegRNA35-6 showed up to 20% of the desired modification when a second nick was orchestrated at the position +57 from the original nick site. For the three pegRNAs, there was no increase using the PE3b strategy when the second nick was at position −24. These experiments were carried out in five independent replicates (*n* = 5).

To further improve the prime editing efficiency, we tested the PE3 approach previously used in the Anzalone et al. article [9]. The PE3 uses an additional sgRNA that nicks the genome at the opposite stand of the first pegRNAs induced nick. The distance between the second sgRNA nick and the first pegRNA nick site can vary depending on the PAM availability. Generally, the improvement is at the optimal distance between 40 and 150 nucleotides. To test the PE3 strategy, we selected the *DMD* gene exon 35 and constructed a modified pU6-pegRNA-GG-acceptor plasmid. The new plasmid contained an additional U6 promoter that permitted the expression of the second sgRNA. We designed two sgRNAs, which were tested with three pegRNAs (i.e., pegRNA35-4, pegRNA35-5 and pegRNA35-6). These two sgRNAs were able to target at −24 and +57 nucleotides from the original nick site of the *DMD* gene exon 35 (Figure 5A). Both the pCMV-PE2 and one of the new pU6-pegRNA-GG-acceptor plasmids were transfected in the HEK293T cells. Three days after the transfection, cells were collected, and the desired modifications were analyzed by Sanger sequencing and the EditR online program. The negative control was only 1% (Figure 5B). A significant increase up to 20% for the desired edit was observed with pegRNA35-4 when the second sgRNA targeted at +57 nucleotides from the first nick site. However, there was no improvement with pegRNA35-5 either at −24 or +57 nucleotides from the original nick site. No improvement was also observed at −24 with pegRNA35-4 and pegRNA35-6 (Figure 5B). These results confirm the hypothesis that the PE3 strategy might be strongly influenced by the distance between the nick induced by the pegRNAs and the sgRNA, and by the RTT and PBS lengths.

Using a sgRNA targeting the sequence mutated by the pegRNA: the PE3b method.

The PE3b strategy aimed to use a sgRNA, which targets the newly synthesized DNA strand containing a nucleotide modified by the pegRNA. The pU6-pegRNA-GG-acceptor plasmid was modified to add an additional U6 promoter able to drive the expression of the sgRNA targeting the sequence resulting from the pegRNAs edit. The sgRNA sequence contained a thymine (T) nucleotide that permitted to bind to the nucleotide sequence including the complementary codon to TGA induced by the pegRNA (Figure 5A). We transfected HEK293T simultaneously with pCMV-PE2 plasmid and the modified pU6-pegRNA-GG-acceptor plasmid coding for the pegRNAs and the sgRNA. Three days later, results showed a decreased percentage of modification with the three pegRNAs tested (pegRNA35-4, pegRNA35-5 and pegRNA35-6) (Figure 5B). Thus, the PE3b strategy did not improve the intended nucleotide mutation for that site.

#### 2.2.3. Modification of the PAM Sequence

**Figure 6:**
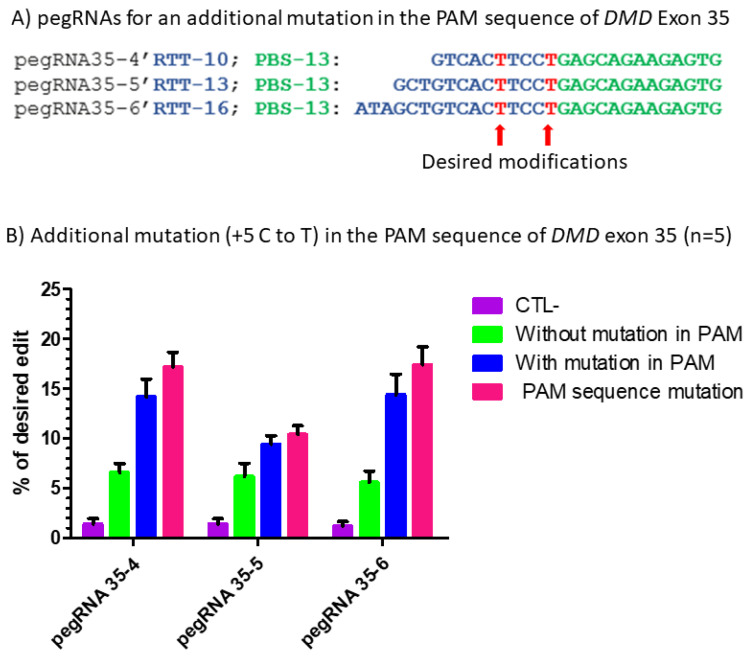
Prime editing of DMD exon 35 with an additional mutation in the PAM sequence. (**A**) The sequences of the Primer Binding Site (PBS, in green) and the Reverse Transcriptase Template (RTT, in blue) of the three pegRNAs targeting DMD exon 35. The T in red in the RTT represents the desired nucleotide to be inserted in the strand not containing the PAM sequence (i.e., in the sense strand in this case) to transform the arginine codon (CGA) into a stop codon (TGA). (**B**) Results of Sanger sequencing and EditR analysis of prime editing of the DMD exon 35 with a modification in the PAM. There was a 1% background level of thymidine (T) in the position of the targeted nucleotide. The pegRNA35-4′ showed a significant reduction (only 1%) in the editing percentage of the arginine codon (CGA) into a stop codon (TGA). The pegRNA PAM (5′AGG3′) was modified to a 5′AAG3′ in 2% of the sequence. The pegRNA35-5′ and pegRNA35-6′ induced, respectively, 9 and 14% C to T to create the desired stop codon (TGA) in exon 35 of the *DMD* gene. The pegRNA35-5′ and pegRNA35-6′ also induced, respectively, 10 and 15% G to T modification, changing the AGG PAM sequence into an AAG sequence, which is not a PAM. These experiments were carried out in five independent replicates (*n* = 5).

Although the repeated transfection and PE3 strategy improved the prime editing efficiency to modify specific nucleotides in the *DMD* gene, the targeted nucleotide mutations remained low. Following these experiments, we thought that if we limited the ability of the SpCas9n-RT to nick the already edited gene sequence, we could obtain better results. In fact, a modification of the PAM sequence recognized by SpCas9n-RT would not allow this enzyme to nick the targeted DNA a second time. For this experiment, we selected pegRNA35-4, pegRNA35-5 and pegRNA35-6 targeting *DMD* gene exon 35 based on the previous assays (see Figure 2B). We modified these pegRNAs to produce pegRNA35-4′, pegRNA35-5′ and pegRNA35-6′ to permit the introduction of an additional mutation in the AGG PAM sequence. However, the second mutation induced by these modified pegRNAs of the AGG PAM sequence in the anti-sense strand into a non-PAM sequence (AAG) also modified the threonine codon (ACC) in the sense strand in a synonymous threonine codon (ACT). HEK293T cells were simultaneously transfected with the pCMV-PE2 plasmid and the new pegRNA plasmids, each containing an RTT sequence with the desired edit and an additional edit to mutate the NGG PAM sequence (Figure 6A). Cell samples were harvested three days later. Exon 35 of the *DMD* gene was PCR amplified, sequenced with the Sanger method, and analyzed by the EditR program. There was only 1% of T in the cytidine targeted site in the negative control cells. The percentage of C to T mutations in the targeted arginine codon (CGA) ranged from 5 to 6% for the different pegRNAs inducing a stop codon (TGA) mutation (Figure 6B). For the pegRNAs (i.e., pegRNA35-4′, pegRNA35-5′ and pegRNA35-6′) containing an additional mutation in the PAM used by the pegRNA, there was no significant difference between the pegRNA35-4′ and the negative control. However, the percentage of desired modification with pegRNA35-5′ and pegRNA35-6′ containing an additional mutation in the PAM increased to 9 and 14%, respectively. These results suggest that altering the PAM sequence improves the prime editing efficiency but only for some pegRNAs (Figure 6B). The mutation introduced in the PAM sequence was as high as the initial edit inserting a stop codon (TGA), with the PAM editing percentage ranging from 10 to 15% for pegRNA35-5′ and pegRNA35-6′ (Figure 6B).

#### 2.2.4. Combination of the PE3 Method and the pegRNA PAM Mutation

**Figure 7:**
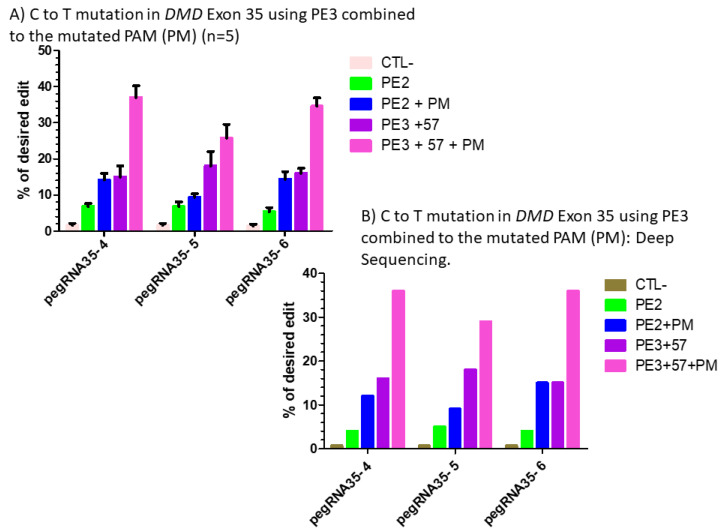
(**A**) prime editing of DMD exon 35 using the PE3 strategy combined with the mutation in the PAM (PM). There was a 1% background level of thymidine (T) in the position of the targeted nucleotide. However, the combination of PE3 using a sgRNA to induce a nick at +57 nucleotides from the pegRNA nick site with the mutation of the PAM sequence showed a significant increase from 6% with the PE2 technique to 38% with pegRNA35-4′, from 5% to 30% with pegRNA35-5′ and from 4% to 39% with pegRNA35-6′. The experiments were carried out in five independent replicates (*n* = 5). (**B**) prime editing of DMD exon 35 using the PE3 strategy combined with the mutation in the PAM (PM): deep sequencing results. There was a 1% background level of thymidine (T) in the position of the targeted nucleotide. However, the combination of PE3 using a sgRNA to induce a nick at +57 nucleotides from the pegRNA nick site and the mutation of the PAM sequence showed a significant increase from 4% with the PE2 technique to 38% with pegRNA35-4′, from 5% to 29% with pegRNA35-5′ and from 4% to 36% with pegRNA35-6′. This deep sequencing experiment was done only once (*n* = 1).

Since we obtained a significant improvement using the PE3 approach with pegRNA35-6 and a sgRNA inducing a nick at +57 nucleotides from the pegRNA nick site, we decided to experiment with the same approach when the additional mutation is introduced in the pegRNA PAM sequence (5′AGG 3′). We used the same pegRNAs (pegRNA35-4′, pegRNA35-5′ and pegRNA35-6′) previously synthesized to introduce a mutation in the pegRNA PAM sequence (Figure 6A). We also used the same sgRNA previously used in the PE3 experiment that nick at +57 nucleotides from the original nick site (Figure 5A). Results showed that the level of negative control remained at 1% (Figure 7A). However, we observed up to 38% increase with the three pegRNAs, suggesting that an additional mutation in the NGG PAM sequence permitted an increase of 1.6- to 2.5-fold of the initial PE3 results (Figure 7A). Thus, an additional mutation in the PAM sequence permitted an increase by 2- to 3-fold of the initial introduction of a stop obtained using PE2 strategy (Figure 7A). This strategy indicates that the prime editing efficiency could be significantly improved by adding an additional modification in the pegRNA PAM. However, this approach may sometimes modify an amino acid codon, which fortunately was not the case for this experiment.

#### 2.2.5. Deep Sequencing Analyses

To confirm and validate the Edit R approach used to estimate the editing percentage, we selected 18 amplicons from one of the replicates of DMD exon 35 and sent them to the Genome Quebec Innovation Centre at McGill University for deep sequencing using the illumina technology. Compared to Sanger sequencing, this technology permits obtaining about 10,000 reads. Results showed 1% background and, respectively, 4%, 5% and 4% C to T modifications with pegRNA35-4, pegRNA35-5 and pegRNA35-6 using PE2 approach (Figure 7B). We observed 12%, 9% and 16% C to T modifications, respectively, with pegRNA35-4, pegRNA35-5 and pegRNA35-6 using the PE2 approach with a mutation in the PAM sequence (PM). We obtained 16%, 18% and 15% C to T modifications with pegRNA35-4, pegRNA35-5 and pegRNA35-6 using the PE3 approach. We also obtained 38%, 29% and 36% C to T modification with pegRNA35-4, pegRNA35-5 and pegRNA35-6 using the PE3 approach with a mutation in the PAM sequence (PM) (Figure 7B). Thus, this approach permitted an average increase in the editing efficiency of 8-fold.

**Figure 8:**
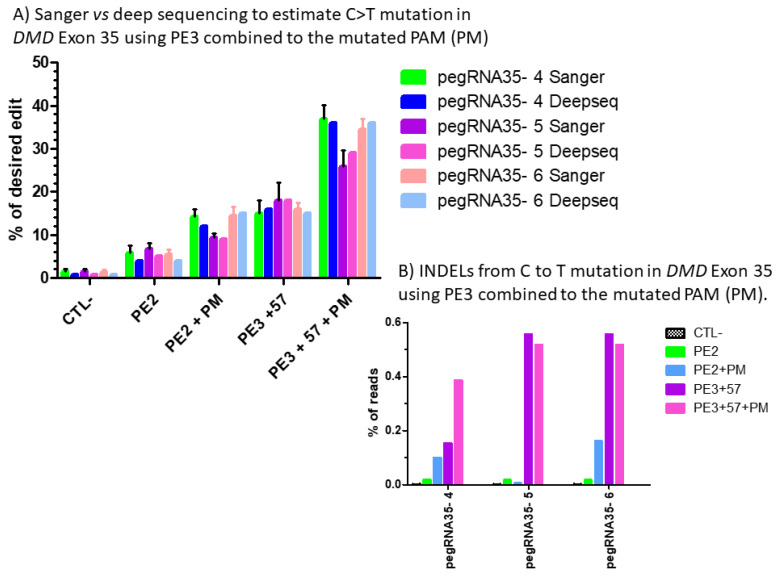
(**A**) Sanger vs. deep sequencing to estimate the percentage of C to T mutations in DMD exon 35 using PE3 combined with the mutated PAM (PM). The percentages of modification estimated by the Edit R program using the average Sanger sequencing results were not different for those obtained with CRISPRESSO using the Illumina deep sequencing results for the pegRNA 4, pegRNA 5 and pegRNA6. (**B**) Indel level from prime editing of DMD exon 35 using the PE3 strategy combined with the mutation in the PAM (PM): deep sequencing results. There were no Indels in the control sample. The Indel levels ranged from 0 to 0.16 and from 0.1 to 0.56% for PE2 and PE3 strategy, respectively, using pegRNA35-4, pegRNA35-5 and pegRNA35-6.

These results confirmed the previous results obtained with the Sanger sequencing and the Edit R analyses (Figure 8A). Thus, the EditR analysis can be used as a rapid screening to detect point mutations produced with the prime editing technology. The deep sequencing results analyzed with the CRISPRESSO online program [14] showed low Indel levels ranging from 0 to 0.56% (Figure 8B).

### 2.3. Correction of Point Mutation in Human Myoblasts from a DMD Patient

**Figure 9:**
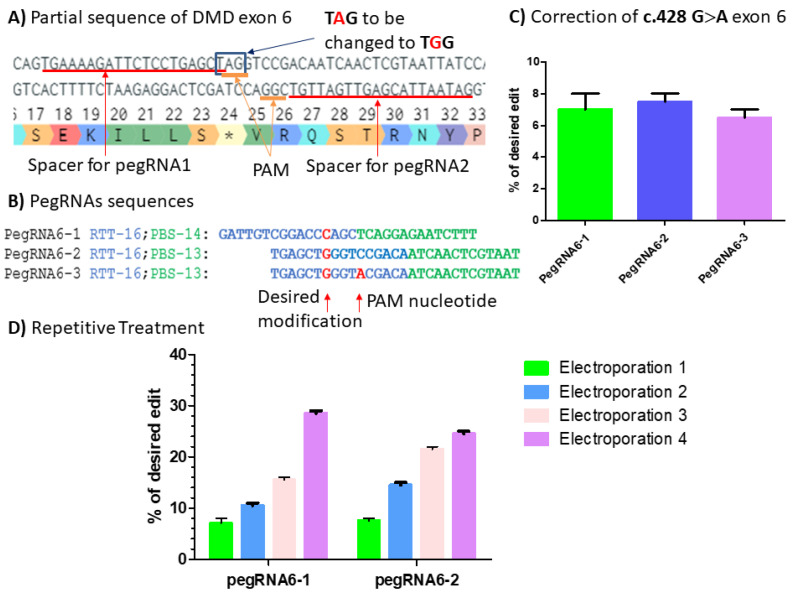
(**A**) Partial sequence of DMD exon 6 carrying c.428 G>A point mutation (*). The red lines indicate the spacer sequences for pegRNAs 1, 2 and 3 preceded by the PAM sequences AGG in the upper strand and CGG in the bottom strand. The blue square indicates the TAG codon to be changed to TGG codon, which is the tryptophan (W) amino acid. (**B**) PegRNA sequences with intended modifications highlighted in red: the C nucleotide to be introduced by pegRNAs6-1 and the G nucleotide to be introduced by pegRNA6-2 and pegRNA6-3. The A nucleotide is introduced by pegRNA6-3 for the simultaneous modification of the PAM sequence. (**C**) Results from Sanger sequencing for the three pegRNAs. (**D**) Sanger sequencing results for pegRNA6-1 and 2 for four repetitive treatments. The experiments were carried out in independent triplicates (*n* = 3).

Human myoblasts were obtained from a skin biopsy from a DMD patient carrying the c.428 G>A point mutation in exon 6 of the *DMD* gene. We identified two PAM sequences around the desired mutation to change A to G (Figure 9A). The PAM in the upper strand of the DNA was considered for the design of pegRNA6-1 to make the correction at the position +4 from the nick site (Figure 9A,B). The PAM sequence at this position could not be modified for prime editing optimization because it was not possible to produce a silent mutation. The other PAM sequence located in the bottom strand of the DNA sequence was considered for the design of pegRNA6-2 and pegRNA6-3 to make the same modification at position +10 from the nick site (Figure 9A,B). The pegRNA6-3 had the same sequences as pegRNAs6-2 but also included a mutation in the PAM sequence to change CGG to CGT. Two additional sgRNAs targeting at +60 and +57 for PE3 were also designed to be used with pegRNA6-1, which used a PAM in the upper strand, and pegRNA6-2 and pegRNA6-3, which used a PAM in the bottom strand. A total of 2 µg of plasmids (1 µg of PE2 plasmid and 1 µg of different pegRNA plasmids) were electroporated into human myoblasts carrying the targeted point mutation using the Neon™ Transfection System. The electroporated cells were harvested three to six days after the treatment and split into two parts. One part of the harvested cells was used for DNA extraction, amplification, and sequencing of a specific sequence of DMD exon 6 carrying the mutation. Results showed up to 8% corrections using pegRNA6-1 and pegRNA6-2 (Figure 9C). PegRNA6-1 and pegRNA6-2 were subsequently chosen for four repetitive treatments, which produced up to 29% and 25% mutations, respectively, for the pegRNA6-1 and pegRNA6-2 (Figure 9D).

**Figure 10:**
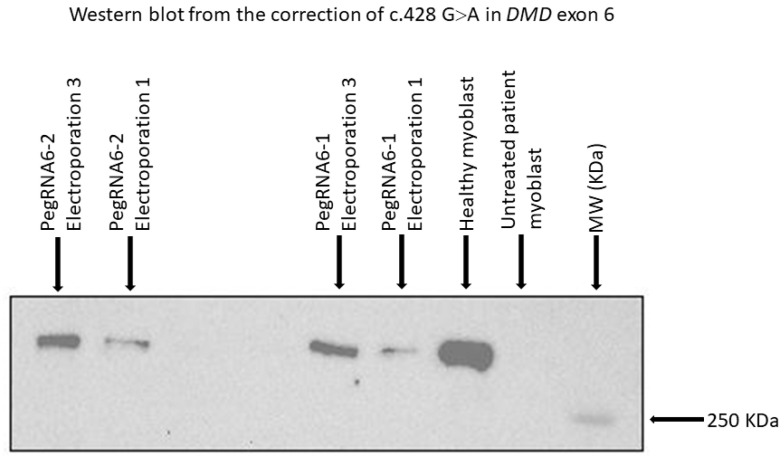
Western blot results from repetitive treatments (electroporations 2 and 3) showing dystrophin expression with pegRNA6-1 and pegRNA6-2. Ctrl+ and Ctrl− indicate, respectively, positive results (myoblasts without a DMD mutation) and negative controls (untreated DMD myoblasts used for the correction using prime editing).

The other part of the harvested cells was expanded during three to six days after electroporation to allow the fusion of myoblasts to make myotubes to be used for Western blot analysis to verify the dystrophin expression. This was carried out during the repetitive treatment steps. Western blot results from treatments 1 and 3 (Figure 9D) showed the dystrophin expression compared to untreated cells (Figure 10).

## 3. Discussion

Our results confirm those of Anzalone et al. [9], i.e., that the prime editing technology may be used to induce specific nucleotide mutations. This is very encouraging for the possibility of using this technique for the correction of point mutations responsible for hereditary diseases. However, our initial experiments were carried out in proliferating cell lines. Thus, in future experiments, we will have to confirm that specific nucleotide mutations could be induced in vivo directly in the muscle fibres that do not proliferate [15]. This will have to be tested in a mdx mouse model of DMD.

The fact that the percentage of nucleotide mutations increased with repeated prime editing treatment, PE3 strategy and mutations in the pegRNA PAM sequence is very encouraging. These percentages might be significantly improved by adapting recently developed Prime editing optimizations [16,17]. It is important to note that up to 8 and 28% *DMD* c.428 G>A point mutation corrections were obtained in the *DMD* gene leading to dystrophin expression after the fusion of treated myoblasts. Muscle fibres contain thousands of nuclei (i.e., up to 60 nuclei per mm). The nuclear domain [18] (i.e., the length of muscle fibre membrane over which the dystrophin is present when there is only one competent nucleus) is about 439 μm [19]. Since there are about 30 nuclei in the nuclear domain, this means that the modification of the *DMD* gene in 1 out of 30 nuclei, i.e., only 3% of the *DMD* genes, would be sufficient for the expression of dystrophin over most of the muscle fibre membrane and thus sufficient to produce a therapeutic effect. Thus, the percentage of nucleotide modifications induced by the prime editing technique is above the minimum required to expect a beneficial outcome, if such a percentage may be obtained in muscle fibres in vivo.

It is clear that for the prime editing technique, several different pegRNAs have to be tested for each intended mutation since some pegRNAs did not work at all. For each intended mutation, it may be necessary to test several methods: different pegRNAs having PBS and RTT sequences of various lengths, repeated treatments, modification of the pegRNA PAM and insertion of a second nick at different distances for the pegRNA nick. Additional research will have to be done to understand the reason for poor and good results to improve the design of the pegRNAs. Moreover, some genes, like EMX1, seem to be more easily edited. The repeated administration of some pegRNAs did not increase the percentage of gene correction while there was a good improvement for other pegRNAs, and we have to understand why. The percentage of specific nucleotide mutations in the EMX1 gene also increased with a second administration reaching 49%, however, the percentage of corrections did not increase further with a third treatment. Again, this phenomenon has to be understood. The repetitive treatments give an advantage for the creation of cell lines for different hereditary diseases. It might be possible to get 100% modification for some easy to modify genes or a high percentage to facilitate the cloning to obtain a cell line with 100% modifications.

Chemello et al. [20] recently used the Prime editing technique for the reframing of exon 52 in iPSC-derived cardiomyocytes carrying a *DMD* del51 mutation by inserting two nucleotides (AC) in exon 52. Both their article and our current results provide evidence that specific nucleotides of the *DMD* gene may be modified by the Prime editing technology. However, many additional experiments will be required before such nucleotide mutations can be attempted in a clinical trial.

## 4. Materials and Methods

### 4.1. Plasmid

PRIME Editor 2 (PE2) plasmid coding for the SpCas9 nickase gene fused with the MLV reverse transcriptase was obtained from Addgene Inc. (Watertown, MA, USA) (pCMV-PE2 # 132775). The plasmid used to express a pegRNA was also obtained from Addgene Inc. (pU6-pegRNA-GG-acceptor # 132777). Cloning of pegRNA into the BSAI-digested pU6-pegRNA-GG-acceptor was as described by Anzalone et al. [9]. Oligonucleotides used to construct all pegRNAs were obtained from Integrated DNA technologies Inc. (Coralville, IO, USA).

### 4.2. Cell Culture

HEK293T grew in DMEM-HG medium (Wisent Inc., St-Bruno, QC, Canada) supplemented with 10% FBS (Wisent Inc., St-Bruno, QC, Canada) and 1% penicillin-streptomycin (Wisent Inc., St-Bruno, QC, Canada) at 37 °C and with 5% CO_2_, in a humidified incubator. The day before transfection, cells were detached from the flask with a Trypsin-EDTA solution (Sigma Inc. Hong Kong, China) and counted. Cells were plated in a 24-well plate at a density of 60,000 cells per well with 1 mL of culture medium. On transfection day, the medium was replaced with 500 μL of fresh medium. Cells were transfected with 1 μg of total DNA (500 ng of each plasmid when co-transfection was required) with Lipofectamine 2000 (Invitrogen™ Inc. Carlsbad, CA, USA) according to the manufacturer’s instructions. The medium was changed to 1 mL of fresh medium 24 h later and cells were maintained in incubation for 72 hrs before genomic DNA extraction. For multiple treatment experiments, cells were plated on day 0 and transfected as described above. On day 3, 50% of cells were recovered for genomic DNA extraction and the remaining 50% were plated in one well of a 6-well plate containing 2 mL of fresh culture medium. The plate was put back into the incubator and the cells were allowed to grow until day 6. Cells were detached from the well with trypsin-EDTA solution, counted and DNA was extracted from cell samples. The remaining cells were plated at 60,000 cells per well in a 24-well plate. The following day, cells were transfected as described above. We used the same procedure for the third treatment.

The human myoblasts were grown in a homemade medium containing 4 volumes of DMEM-HG medium for 1 volume of medium 199 (Invitrogen™ Inc., Carlsbad, CA, USA) supplemented with Fetuin 25 µg/mL (Life Technologies, Carlsbad, CA, USA), hEGF 5 ng/mL (Life Technologies, Carlsbad, CA, USA), bFGF 0.5 ng/mL (Life Technologies, Carlsbad, CA, USA), Insulin 5 µg/mL (Sigma-Aldrich Canada Co, 91077C-1G, Oakville, ON, Canada), Dex 0.2 µg/mL (Sigma-Aldrich Canada Co., Oakville, ON, Canada). A total of 2 µg of plasmids (1 µg of pCMV-PE2 plasmid, 1 µg of pU6-GG-acceptor plasmid containing the pegRNA sequence and the sgRNA for PE3) were mixed to 100,000 human myoblasts and electroporated with the Neon Transfection System following the program 1100 volt/20 ms/2 pulses. These electroporated cells were placed in one well of a 24-well culture plate containing 500 µL of the home made medium. The electroporation medium was changed to 1 mL fresh medium after 24 h and cells were detached with trypsin and transferred into 1 mL culture medium over the next 48 h. Half of the harvested cells were used for DNA extraction and the remaining volume was transferred to one well of a 6-well culture plate containing 2 mL of the homemade medium. At 80 to 90% confluency, the medium was changed to 2 mL DMEM containing 1% FBS to induce myoblast fusion to form myotubes, which were harvested a few days later for Western blot analysis of dystrophin.

### 4.3. Genomic DNA Preparation and PCR Amplification

HEK293T cells were detached from wells directly by up and down pipetting of the culture medium and were transferred in 1.5 mL Eppendorf tubes. Human myoblasts were detached using Trypsin-EDTA solution (Sigma-Aldrich Canada Co., Oakville, ON, Canada) and were collected in 1 mL of the original medium. HEK293T cells or human myoblasts were spun for 5 min at 9000 RPM in a microcentrifuge at room temperature. Cell pellets were washed once with 1 mL of PBS 1X and spun again for 5 min at 9000 RPM. Genomic DNA was prepared using the DirectPCR Lysis Reagent (Viagen Biotech Inc., Los Angeles, CA, USA). Briefly, 50 μL of DirectPCR Lysis Reagent containing 0.5 μL of a proteinase K solution (20 mg/mL) was added to each cell pellet and incubated overnight at 56 °C followed by another incubation at 85 °C for 45 min and centrifugation at 13,000 RPM for 5 min. Then, 1 μL of each genomic DNA preparation (supernatant) was used for the PCR reaction for each primer set (Table 1). PCR temperature cycling was as follows: 98 °C: 30 s and 35 cycles of 98 °C: 10 s, 60 °C: 20 s, 72 °C: 45 s. A final extension at 72 °C for 5 min was also performed. We used Phusion^TM^ High-Fidelity DNA polymerase from Thermo Scientific Inc. (Waltham, MA, USA) for all PCR reactions. Finally, 5 μL of amplicons was electrophorized in 1X TBE buffer on 1% agarose gel to control the PCR reaction qualities and to make sure that only one specific band was present. The remaining 45 μL PCR reaction was Sanger sequenced with an internal primer (Table 1). Sanger sequencing results were analyzed with the EditR online program (https://moriaritylab.shinyapps.io/editr_v10/ (accessed on 1 May 2022)).

### 4.4. Deep Sequencing Analysis

Deep sequencing samples were prepared by a PCR reaction (as described above) with special primers containing a bar code sequence (BCS) to permit the subsequent deep sequencing (Table 1). PCR samples were sent to Genome Quebec Innovation Centre at McGill University to sequence amplicons with the Illumina sequencer. Roughly 6000–10,000 reads were obtained per sample. Illumina sequencing results were analyzed with the CRISPRESSO 2 online program (https://crispresso.pinellolab.partners.org/ (accessed on 1 May 2022)) [14].

### 4.5. Western Blot Analysis

Myotubes were detached directly from a culture plate with 400 μL of lysis buffer supplemented with protease inhibitors. Then, 1 µL of extracted proteins and different concentrations of BSA (used as standard) were put on nitrocellulose membrane and colored with amino black 10B. The membrane was scanned by ChemiDoc XRS + system (Bio-Rad Laboratories Inc., Hercules, CA, USA) and quantified using Image Lab 6.0.1 software (Bio-Rad Laboratories Inc., Hercules, CA, USA) according to the manufacturer’s instructions. A total of 20 µg of extracted protein samples was separated by SDS-PAGE (Bio-Rad Inc., 4–7%) and transferred onto a nitrocellulose membrane. A mouse monoclonal antibody against dystrophin (clone MANDYS8, ABNOVA Inc., Taipei, China) was used for immunoblotting analysis. HRP conjugated goat anti-mouse (Thermo Scientific Inc., Waltham, MA, USA), was used as secondary antibody. The membrane was developed using Clarity^TM^ Western ECL substrate (Bio-Rad Laboratories Inc., Hercules, CA, USA) and scanned by ChemiDoc XRS + system (Bio-Rad Laboratories Inc., Hercules, CA, USA).

### 4.6. Statistical Analysis

Data were analyzed using the Graph Pad PRISM 5.0 software package (Graph Pad Software Inc., La Jolla, CA, USA). The comparisons between the mean editing percentage amongst different groups were performed using the Mann–Whitney’s nonparametric *U* test. A *p*-value < 0.05 was considered statistically significant for a 5% confidence interval.

## Figures and Tables

**Table 1:** Primer sequences for PCR, Sanger and deep sequencing.

Primer Nomenclature	Primer Sequence
Sanger sequencing of DMD exon 9 Rev	ACAAACCAGCTCTTCACGAGG
Sanger sequencing of DMD exon 35 Fwd	ATTACTTGAAGGTCAATGCTCTCC
Sanger sequencing of DMD exon 20	AGAGTTCCAAAGTGAGAGGCC
Sanger sequencing of DMD exon 43	AGGATCCAGCAAAGGAAAGCAG
Sanger sequencing of DMD exon 55	TGCCTTCCCCCATACAAACGC
Sanger sequencing of DMD exon 61	GTGGCTGCCAATGAGTTGACTG
PCR of DMD exon 20 Fwd	TCATGCAGCCTTCCAGCTCC
PCR of DMD exon 20 Rev	CCAAGCTTGTTGTTGACCCGTG
PCR of DMD exon 43 Fwd	AGGAGATGTCCGGGCTTGAG
PCR of DMD exon 43 Rev	AGCACCTCAATGCCCCAATCTG
PCR of DMD exon 55 Fwd	GAAACCTCCTCTGTGGAGAGG
PCR of DMD exon 55 Rev	TCTCCTTGACCGAAGCTCTGG
PCR of DMD exon 61 Fwd	GTCACCTATACCAAATGTCACC
PCR of DMD exon 61 Rev	CTCCTGAGCAAAGTGTTCCAC
PCR of DMD exon 9 Fwd	GCCGGATTGAAGAGTACCATCC
PCR of DMD exon 9 Rev	GCAGCCTTTGTCAAAGAGAACC
PCR of DMD exon 6 Fwd	CACTGAAGATCAAGGACATTC
PCR of exon 6 Rev	CATCAGAGTCTAAATCACCACT
PCR of DMD exon 35 Fwd	AGAGACATACCATGGCATTATTGG
PCR of DMD exon 35 Rev	ATATCTGCCTTTAGCCACTACATG
Deepseq of DMD exon 35 Fwd	**ACACTGACGACATGGTTCTACA**ATTACTTGAAGGTCAATGCTCTCC
Deepseq of DMD exon 35 Rev	**TACGGTAGCAGAGACTTGGTCT**TCGGTCGTATATGCATCTTAACTC

## Data Availability

The data presented in this study are available on request from the corresponding author.
